# Electronic cigarette aerosols disrupt airway barrier function via MMP-dependent E-cadherin cleavage: findings from cell culture and murine models

**DOI:** 10.1007/s00204-026-04456-2

**Published:** 2026-06-05

**Authors:** Amelia L. Beaumont, Sarah G. Ozeki, Claire E. Lee, Robert L. Chatburn, Russell P. Bowler, Fariba Rezaee

**Affiliations:** 1 Department of Inflammation and Immunity, Cleveland Clinic Research, Lerner Research Institute, Cleveland Clinic Foundation, 9500 Euclid Ave. NB20, Cleveland, OH USA; 2 Enterprise Respiratory Care Research Cleveland Clinic Foundation, Cleveland, OH USA; 3 Genomic Sciences and Systems Biology, Cleveland Clinic Research, Cleveland Clinic Foundation, Cleveland, OH USA; 4 Center for Pediatric Pulmonary Medicine, Cleveland Clinic Children’s, Cleveland, OH USA

**Keywords:** Electronic cigarette, Vaping, Respiratory syncytial virus (RSV), Airway epithelial cells, Epithelial barrier

## Abstract

Electronic cigarette (e-cigarette) use continues to rise, yet the toxicological mechanisms by which inhaled aerosols impair airway epithelial integrity remain poorly defined. E-cadherin, a key adherens junction protein required for epithelial cohesion, can undergo proteolytic cleavage to generate soluble E-cadherin (sE-cad), a mediator of epithelial barrier dysfunction and inflammation. We hypothesized that e-cigarette aerosol exposure promotes MMP-dependent E-cadherin cleavage, resulting in sE-cad release and epithelial barrier disruption. Using differentiated primary normal human bronchial epithelial (NHBE) cells, 16HBE cells, and a murine whole-body exposure model, we examined epithelial injury induced by physiologically relevant aerosols generated with a programmable puffing system. Air Factory salt e-liquid was used at 18 mg/mL nicotine for in vitro studies and 36 mg/mL for in vivo exposures. Immunoblotting demonstrated increased sE-cad levels in apical supernatants and bronchoalveolar lavage fluid. Aerosol exposure upregulated MMP-2, MMP-9, and MMP-12 expression. E-cigarette aerosol exposure significantly reduced transepithelial electrical resistance, increased FITC-dextran permeability, and disrupted airway epithelial barrier structure, indicating impaired barrier integrity and function. These effects were attenuated by pretreatment with the MMP inhibitor fisetin, which preserved barrier function and junctional protein localization. Fisetin treatment significantly reduced e-cigarette–induced sE-cad release and was associated with a marked reduction in MMP-9 expression, whereas MMP-2 and MMP-12 levels were not altered, identifying MMP-9 as a key mediator of E-cadherin cleavage. Collectively, these findings establish MMP-dependent E-cadherin cleavage as a mechanistic driver of e-cigarette–induced epithelial barrier dysfunction and identify sE-cad as a potential early biomarker and therapeutic target for aerosol-induced airway injury.

## Introduction

Tobacco remains the leading cause of preventable death in the United States, responsible for nearly half a million deaths annually (CDC 2024). The rapid rise of electronic cigarette (e-cigarette) use, particularly among adolescents, has created a significant toxicological and public health concern. First introduced in 2007 as smoking cessation devices (Zhu et al. 2014), e-cigarettes are now widely available in youth-appealing flavors (Ma et al. 2022). According to the 2024 National Youth Tobacco Survey, 2.25 million U.S. youth reported using tobacco in the past 30 days, including 1.63 million e-cigarette users—88% of whom preferred flavored products (FDA 2024). Despite their widespread use, the mechanisms by which inhaled e-cigarette aerosols exert toxic effects on the airway epithelium remain incompletely understood.

Emerging evidence indicates that e-cigarettes compromise airway epithelial integrity, an effect of direct toxicological relevance. In vitro studies demonstrate that e-cigarette aerosols reduce transepithelial electrical resistance (TEER) and alter the localization of apical junctional complex (AJC) proteins (Raduka et al. 2023). In vivo, aerosol exposure disrupts epithelial architecture in murine models (Beaumont et al. 2025). E-cigarette use has also been associated with increased susceptibility to respiratory viral infections, including SARS-CoV-2, human rhinovirus, and RSV (Gaiha et al. 2020; Raduka et al. 2023; Rebuli et al. 2021; Wu et al. 2014), suggesting impaired barrier defenses against inhaled toxicants and pathogens.

The airway epithelium is a primary target for inhalation toxicology, functioning as a selective barrier against environmental particles, microbes, and chemical toxicants. This barrier depends on TJs and AJs, which regulate paracellular permeability and coordinate immune responses (Georas and Rezaee 2014; Rezaee and Georas 2014; Smallcombe et al. 2019). Among these, E-cadherin is critical for maintaining epithelial cohesion. Loss of E-cadherin—through reduced expression or proteolytic cleavage—impairs repair, increases permeability, and amplifies inflammation (Gao and Rezaee 2022; Linfield et al. 2021b).

Insights from viral infection models highlight the toxicological importance of E-cadherin integrity. During RSV infection, proteolytic cleavage of E-cadherin generates soluble E-cadherin (sE-cad), which appears in bronchoalveolar lavage (BAL) fluid and in epithelial cell supernatants (Smallcombe et al. 2019). Functional assays demonstrate that sE-cad destabilizes TJs and weakens barrier defense, acting as an active mediator of epithelial dysfunction. sE-cad fragments can also potentiate injury by enhancing matrix metalloproteinase (MMP) activity (Samuels et al. 2023). Stabilizing E-cadherin reduces epithelial injury in these models (Rezaee and Georas 2014; Rezaee et al. 2017), underscoring its relevance to inhalation toxicology.

Despite evidence that e-cigarettes impair epithelial permeability and disrupt AJCs, the upstream molecular pathways remain poorly defined. No studies have determined whether e-cigarette aerosols induce proteolytic generation of sE-cad or identified the proteases responsible. Drawing from viral models, we hypothesized that e-cigarette aerosols enhance E-cadherin cleavage, increasing sE-cad levels that signal epithelial injury and amplify barrier dysfunction.

In the present study, we used established cell culture systems and murine inhalation models to test this hypothesis and to define the role of MMPs—key mediators of epithelial injury in toxicant exposures. By linking e-cigarette exposure to sE-cad generation and MMP activity, this work provides mechanistic insight into how inhaled aerosols compromise the airway barrier. These findings have direct toxicological relevance by identifying sE-cad as a potential early biomarker of e-cigarette–induced epithelial injury and informing strategies to mitigate toxicant-driven airway damage.

## Materials and methods

### Antibodies

Primary monoclonal (mAbs) and polyclonal (pAbs) antibodies were used for immunoblotting and immunofluorescent staining to identify proteins in the AJC. Primary antibodies include occludin (cat#ab216327, and #33-1500) from Abcam (Waltham, MA) and Invitrogen (Eugene, OR),), β-catenin (cat#ab32572 and #610153) obtained from Abcam (Waltham, MA) and BD Biosciences, E-cadherin (cat#SC-8426, #610181, and #ab40772) obtained from BD Biosciences (Milpitas, CA), Abcam (Waltham, MA), and Santa Cruz Biotechnology (Dallas, TX), zonula occludens-1 (ZO-1) (cat#33-9100 and #40-2200) from Invitrogen (Eugene, OR) and Thermo Fisher (Waltham, MA). MMP-2 (cat#10373-2-AP), MMP-9 (cat#10375-2-AP), and MMP-12 (cat#22989-1-AP) were obtained from ProteinTech (Rosemont, IL). Secondary antibodies—HRP-conjugated goat anti-mouse IgG (cat#31430), HRP-conjugated goat anti-rabbit IgG (cat#31460), Alexa Fluor™ 488 anti-mouse IgG (cat#A21202), Alexa Fluor™ 568 anti-rabbit IgG (cat#A10042), Alexa Fluor 594 Phalloidin (cat#A12381), and Alexa Fluor Phalloidin 488 (cat#A12379)—were obtained from Thermo Fisher Scientific (Waltham, MA) and Invitrogen (Eugene, OR) for western blot (1:5000) and immunofluorescent staining (1:500–1:1000). Detailed information, including clone number, validation, application, and dilution, has been included in the Table 1.

**Table 1:** Primary antibodies used for IF and WB staining

Antibodies	Source	Catalog number	Clone number	Validation	Working dilution
Occludin	Abcam	ab216327	EPR20992	https://doi.org/10.2147/JIR.S331020	1:250 (IF)
Occludin	Invitrogen	33-1500	OC-3F10	https://doi.org/10.1248/bpb.b15-01023	1:300 (IF) 1:500 (WB)
β-catenin	Abcam	ab32572	E247	https://doi.org/10.1002/hep.30270	1:300 (IF)
β-catenin	BD Biosciences	610153	14	https://doi.org/10.1016/j.jbc.2021.100848	1:2000 (WB)
E-cadherin	BD Biosciences	610181	36	https://doi.org/10.1371/journal.pgen.1008451	1:250 (IF); 1:200 (WB)
E-cadherin	Abcam	Ab40772	EP700Y	https://doi.org/10.1371/journal.pone.0022023	1:300 (IF)
E-cadherin	Santa Cruz Biotechnology	Sc-8426	G-10	https://doi.org/10.4081/ejh.2023.3908	1:500 (WB)
GAPDH	Abcam	ab8245	6C5	https://doi.org/10.1038/s41467-018-08187-6	1:50,000 (WB)
ZO-1	Thermo Fisher	40-2200		https://doi.org/10.1186/s12987-022-00371-7	1:1000 (WB)
ZO-1	Invitrogen	33-9100	ZO1-1A12	https://doi.org/10.7554/eLife.65811	1:300 (IF)
Actin	Invitrogen	A12379		https://doi.org/10.7554/eLife.42448	1:1000 (IF)
Cortactin	MilliporeSigma	05-180-I	4F11	https://doi.org/10.1073/pnas.87.9.3328	1:200 (IF)
MMP-2	ProteinTech	10373-2-AP		https://doi.org/10.3390/ijms23031240	1:1000 (IF)
MMP-9	ProteinTech	10375-2-AP		https://doi.org/10.1242/dmm.040972	1:1000 (IF)
MMP-12	ProteinTech	22989-2-AP		https://doi.org/10.1093/ecco-jcc/jjab064	1:1000 (IF)

*IF* immunofluorescence, *WB* Western Blot

### Chemicals

Commercially available unflavored Air Factory electronic liquid (e-liquid) containing nicotine salt (18–36 mg/mL) was purchased from a local vape shop. Different nicotine concentrations were used for in vitro (18 mg/mL) and in vivo (36 mg/mL) exposures to account for differences in aerosol dilution, deposition efficiency, and exposure dynamics between systems, consistent with prior studies using air–liquid interface and whole-body inhalation models (Beaumont et al. 2025; Gaiha et al. 2020; Raduka et al. 2023; Wu et al. 2014). The bioflavonoid fisetin (CAS No. 528-48-3) was obtained from PhytoLab (PhytoLab GmbH & Co. KG, Vestenbergsgreuth, Germany).

### Animals

C57BL/6 mice aged 8–12 weeks, with an average weight of 20 g, were purchased from Jackson Laboratories (Bar Harbor, ME). Mice were housed under standard conditions (12 h light, 12 h dark) and provided with constant access to food. Mice were divided into two groups (10–15 mice per group) and exposed to either e-cigarette aerosols or HEPA-filtered air, weighed daily, and sacrificed at the indicated times. BAL fluid was collected (Linfield et al. 2021a; Smallcombe et al. 2020).

### Human bronchial epithelial cells

16HBE14o- human bronchial epithelial (16HBE) cells were donated by the late Professor Dieter Gruenert, University of California, San Francisco, and are also available commercially through the American Type Culture Collection (ATCC). Cells were cultured in DMEM (Gibco, cat#11995, Waltham, MA) medium with 10% fetal bovine serum (FBS) (Corning, cat#35-011-CV, Durham, NC), HEPES buffer (Gibco, cat#15630, Waltham, MA), and antibiotic–antimycotic (Gibco, cat#15,240–062, Waltham, MA). Cells were grown on rat tail collagen (Corning, cat#354236, Durham, NC)-coated transwell permeable membrane inserts (cat#3470, Corning) as previously described (Raduka et al. 2023). At confluency, the apical media of 16HBE cells were reduced to 30 µL prior to exposure to e-cigarette aerosols.

### Normal human bronchial epithelial cells

Primary normal human bronchial epithelial (NHBE) cells were isolated from the lungs of donors and grown on transwell permeable membranes using PneumaCult Ex Plus Medium (cat#05040) and differentiated under air–liquid interface (ALI) conditions in PneumaCult Ex-Plus ALI Medium (cat#05001). Cells were washed with pre-warmed D-phosphate buffered saline (PBS) (cat#37350) at 2 weeks post-air lift. At full confluency, NHBE cells were exposed to e-cigarette conditions. All NHBE culturing supplies were obtained from StemCell Technologies (Cambridge, MA).

### TEER

TEER was measured using an EVOM2 volt/ohm meter (World Precision Instrument, Sarasota, FL) once 16HBE and NHBE cells reached confluency (Gao et al. 2022; Linfield et al. 2021a; Raduka et al. 2023; Smallcombe et al. 2020). TEER values were shown as a percent change from time zero to 24 h. Experiments began when cell monolayers reached a TEER > 500 Ω × cm^2^.

### Exposure to e-cigarette in vivo

Mice were exposed to aerosolized e-cigarette chemicals using a third-generation e-cigarette device (Joyetech eVic VTC Mini; SCIREQ Inc., Montreal, Canada) attached to an inExpose whole-body exposure chamber for 60 min once daily for four consecutive days. The device was operated at a temperature of 235 °C with a power setting of 70 watts. The e-cigarette device was activated by a stroke controller under the regulation of SCIREQ FlexiWare software (version 6.1). The software simulated a puff profile based on realistic user topography. Wild-type C57BL/6 mice were exposed under a puff profile of 3 puffs/min for 60 min with a puff volume of 70 mL. This profile was selected based on published e-cigarette user topography data and is consistent with profiles used in prior inhalation studies utilizing the SCIREQ inExpose system. It falls within reported human ENDS usage ranges, including puff frequency (2–4 puffs/min), puff duration (2–4 s), and puff volumes (55–100 mL). Similar exposure parameters have been adopted in multiple murine inhalation models (Beaumont et al. 2025; Been et al. 2022; Lee et al. 2018; Robinson et al. 2015, 2016; Wang et al. 2020). Additionally, the cumulative puff count (~ 180 puffs/day) aligns with established human topography data and ensures a consistent and reproducible exposure burden across days (Dawkins et al. 2013). The air pumps were calibrated prior to each exposure using a SCIREQ rotameter. Generated aerosols passed through a condensing chamber into a mixing chamber via pump 4 at a flow rate of 2.0 L/min. The aerosol was mixed and diluted with room-temperature air before entering the whole-body chamber. Following exposure, mice remained in the chamber for 5–10 min to allow aerosol levels to decrease before being returned to their cages for observation. Control mice (air group) were housed in the same room in separate cages to maintain uniform environmental conditions (Beaumont et al. 2025).

### Exposure to e-cigarette in vitro

An ASL 5000 breathing simulator (IngMar Medical, Pittsburgh, PA) was used to generate realistic breathing patterns of patients. Specialized software uses pre-programmed lung models which mimic realistic inspiratory to expiratory time ratios (I:E). The software was programmed to simulate the normal breathing pattern and e-cigarette puffs of 13–18-year-old teenagers. An air-tight cell chamber (Prototype Core, Cleveland Clinic) with a 400 mL volume was connected to the ASL 5000 breathing simulator. To keep the e-cigarette aerosol from entering the ASL 5000, an anesthesia bag in a rigid cylinder (IngMar Auxiliary Gas Exchange Cylinder) was attached between the breathing simulator and the cell chamber. A fourth-generation e-cigarette (Vaporesso XROS set at 1.2Ω, power 11 W/16 W) and HEPA filter were connected via a three-way valve to the air-tight cell chamber. Unflavored AirFactory e-liquid containing 18 mg/mL nicotine, purchased from a local vape shop, was used. Polarized cell culture plates with reduced media (16HBE) or growing at ALI (NHBE) were placed into the air-tight cell chamber. The ASL 5000 breathing simulator was started, and the puff was set to 4 puffs/min lasting for 3 s, with a volume of 100 mL based on previous studies (Raduka et al. 2023). The puff was followed by two breaths at 12 breaths/min for a total of 15 min. Post-exposure, the cell-culture plates were left in the cell chamber for 5 min to equilibrate before returning to the incubator (37 °C, 5% CO_2_). A separate cell group was exposed to HEPA-filtered air under the same puff/breath pattern. Another separate cell group remained in the incubator for the duration of the experiment (control). TEER was measured at time 0 and indicated times post-exposure, normalized to time 0 and compared to control and air groups. Exposures were repeated as indicated and harvested at the end of experiments.

### FITC-dextran permeability assay in vitro

Fluorescein isothiocyanate (FITC)-conjugated dextran 3000 kDa MW (cat#D3305, Invitrogen) was used to evaluate transmonolayer permeability as previously described (Gao et al. 2022; Smallcombe et al. 2020). 16HBE and NHBE cells were grown on transwell inserts (cat#3421, Corning) to confluency. Once confluent, cells were exposed respectively to air or e-cigarette aerosol as previously described. At 27 h post exposure one, cell culture media was removed, and cells were washed twice with PBS and moved to a new plate using sterile forceps. PBS was added to the basal chamber, and FITC-dextran (540 µg/mL in PBS) was added to the apical chamber. The transwells were incubated at 37 °C and samples were collected from the basal chamber. The FlexStation 3 (Molecular Devices, Sunnyvale, CA) was used to measure FITC fluorescence at an excitation wavelength of 485 nm and an emission wavelength of 528 nm. Fluorescence was measured at 3 min and every five minutes following.

### Western blot analysis

Mouse lung tissues were processed for western blot analysis by homogenization using RIPA buffer. Equal amounts of protein from each sample were separated by SDS-PAGE and transferred to polyvinylidene difluoride (PVDF) membranes (Bio-Rad, cat#1704156, Hercules, CA). Membranes were blocked for 1 h at room temperature (RT) with either 5% bovine serum albumin (BSA) in TBST or 5% nonfat dry milk in TBST, followed by overnight incubation at 4 °C with primary antibodies. The following day, membranes were incubated for 1 h at RT with secondary antibodies. Protein bands were visualized using enhanced chemiluminescence (ECL; Thermo Scientific, cat#32106, Waltham, MA or SuperSignal West Femto; Thermo Scientific, cat#34096, Waltham, MA), and images were captured with the ChemiDoc™ Imaging System (Bio-Rad, RRID:SCR_008426, Hercules, CA). Glyceraldehyde-3-phosphate dehydrogenase (GAPDH) served as a loading control. sE-cad fragments were detected in mouse BAL and cell apical supernatant samples by SDS-PAGE and immunoblotting using SuperSignal™ West Pico PLUS Chemiluminescent Substrate (Thermo Fisher Scientific). Densitometric analysis was performed using ImageJ software (RRID:SCR_003070), and protein band intensities were normalized to GAPDH. Detailed information, including clone number, validation, application, and dilution, has been included in Table 1.

### Immunofluorescence labeling and confocal microscopy

Cell monolayers cultured on transwells were fixed in cold methanol or RT 4% paraformaldehyde (PFA) in PBS. PFA (cat#15710) was obtained from Electron Microscopy Sciences (Hatfield, PA). Membranes fixed in PFA were incubated in 0.1% Triton-X in PBS for 3 min at RT to permeabilize the cells then washed 3 times in PBS. Membranes were placed in blocking solution (5% BSA in PBS) for 1 h at RT then incubated in primary antibodies for 1 h at RT. Following incubation, membranes were washed 3 times in PBS then incubated in secondary antibodies for 1 h at RT. Membranes were rinsed 3 times in PBS then stained with DAPI, then mounted with Vectashield anti-fade mounting medium. Membranes were imaged with × 63 objectives with a Leica TCS-SP8-AOBS inverted confocal microscope (Leica Microsystems, Wetzlar, Germany) and processed using Adobe Photoshop.

### Statistical analysis

Data was analyzed using Microsoft Excel and Prism software (GraphPad Prism, RRID:SCR_002798). Results are presented as mean ± standard deviation (SD). Statistical assessment for comparing multiple groups involved using one-way ANOVA when examining more than two groups, followed by the Tukey post hoc test for all experimental groups. T-tests were used to assess the difference between the two groups. Statistical difference was considered when *p* < 0.05 and *p* values were displayed as *(*p* < 0.05), **(*p* < 0.01), ***(*p* < 0.001), ****(*p* < 0.0001), while no significant difference was indicated as *ns*.

## Results

### E-cigarette aerosols increase sE-cad levels across multiple models

Previous studies have shown that cleaved fragments of AJ and TJ proteins can serve as biomarkers in conditions such as viral infections, cancer, and chronic disease, reflecting epithelial injury and barrier disruption (Grabowska and Day 2012; Martin and Jiang 2009; Repetto et al. 2014; Smallcombe et al. 2019; Symowicz et al. 2007). Based on these observations, we focused on sE-cad as a marker of E-cadherin cleavage following e-cigarette aerosol exposure.

**Fig. 1:**
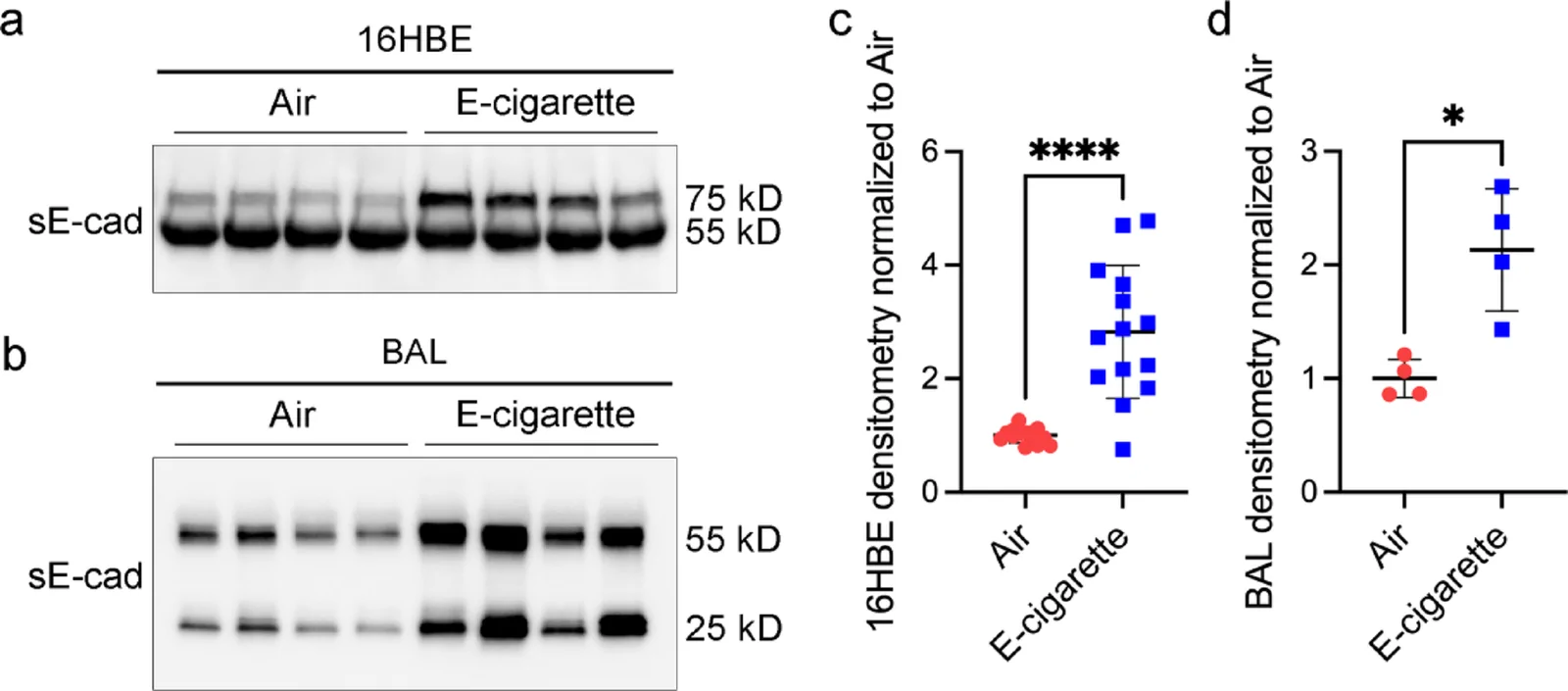
E-cigarette aerosol exposure increases sE-cad across multiple experimental models. Quantitative immunoblotting was used to assess extracellular cleavage of E-cadherin following e-cigarette aerosol exposure. **a** Western blot analysis of apical supernatants from 16HBE cells grown to confluence on transwells and exposed to nicotine-containing e-cigarette aerosol (18 mg/mL) or HEPA-filtered air for 0, 8, and 24 h using the ASL 5000 breathing simulator demonstrated significantly increased sE-cad levels in aerosol-exposed cells. **b** BAL fluid from mice exposed to nicotine-containing e-cigarette aerosol (36 mg/mL) for 4 days showed significantly elevated sE-cad levels compared with air-exposed controls. **c** and **d** Densitometric analysis quantified increased sE-cad levels in both 16HBE apical supernatants and mouse BAL fluid. Data are presented as mean ± SD (n = 4–14 replicates per group). One-way ANOVA with Tukey’s multiple comparisons test. *P < 0.05; ****P < 0.0001

Western blot analysis was performed on apical supernatants collected from 16HBE cells grown to confluence on transwells and exposed to aerosolized e-cigarette aerosol containing nicotine (18 mg/mL) or HEPA-filtered air for 0, 8, and 24 h using the ASL 5000 breathing simulator. Exposure to nicotine-containing aerosols resulted in significantly increased sE-cad levels compared with air-exposed controls (Fig. 1a). Consistent with these in vitro findings, BAL fluid collected from mice after 4 days of exposure to nicotine-containing e-cigarette aerosol (36 mg/mL) demonstrated significantly elevated sE-cad levels relative to air-exposed controls (Fig. 1b). Densitometric analysis confirmed increased sE-cad levels in both 16HBE apical supernatants and mouse BAL fluid (Fig. 1c–d).

Collectively, these results demonstrate that e-cigarette aerosol exposure promotes cleavage of membrane-bound E-cadherin in the airway across both in vitro and in vivo models. These findings further support the use of 16HBE cells as a relevant model for investigating AJC structure and function and for elucidating mechanisms by which e-cigarette aerosols compromise epithelial integrity and barrier function.

### E-cigarette exposure induces increased expression and secretion of MMP-2, MMP-9, and MMP-12

To investigate mechanisms underlying the increased sE-cad levels observed following e-cigarette aerosol exposure, we examined MMP-2, MMP-9, and MMP-12, proteases known to cleave extracellular matrix components and cell adhesion molecules, including E-cadherin, and to contribute to epithelial barrier remodeling and disruption (Grabowska and Day 2012; Lee et al. 2014; Smallcombe et al. 2019).

**Fig. 2:**
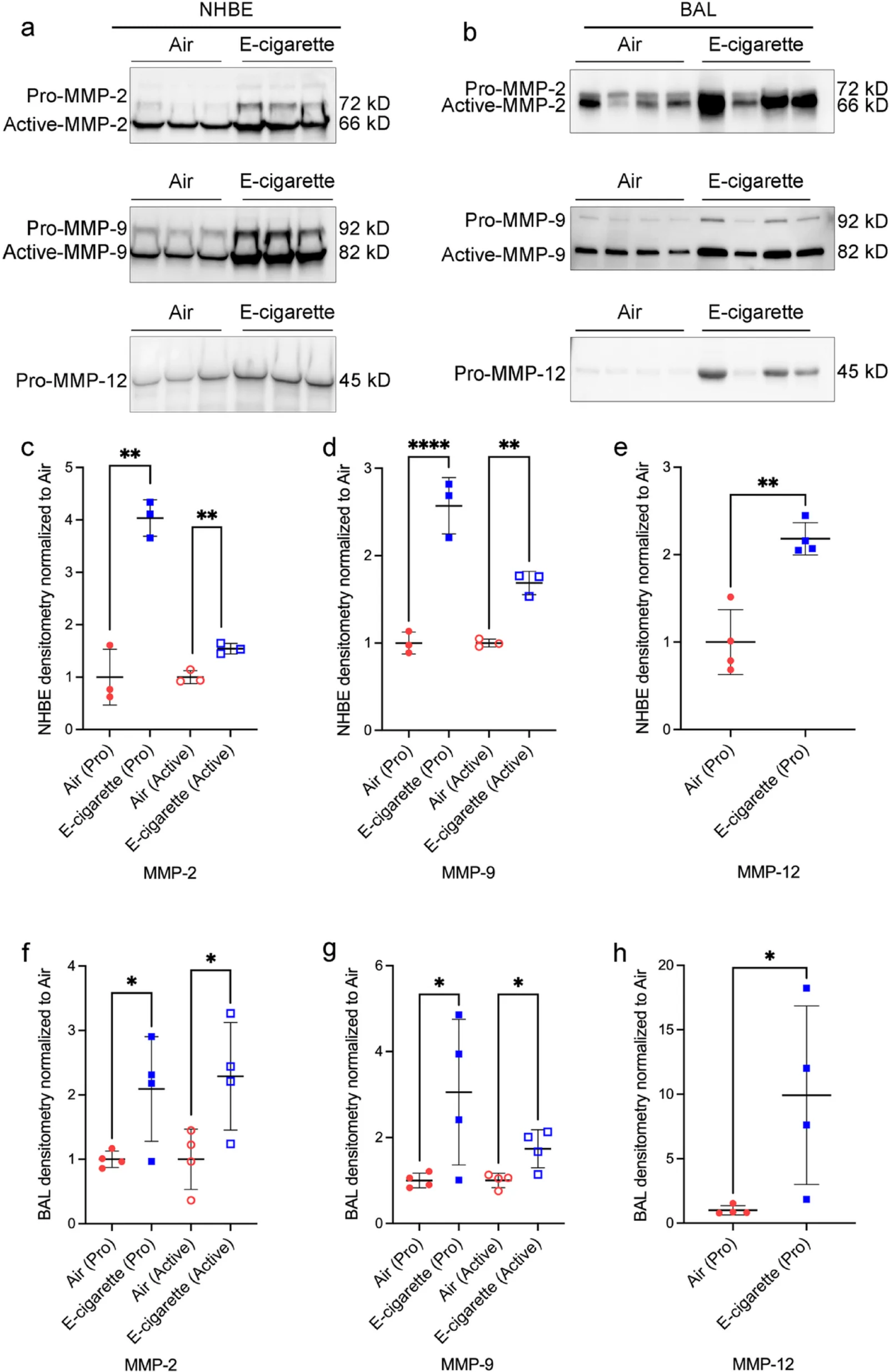
E-cigarette aerosol exposure induces MMP-2, MMP-9, and MMP-12 expression in vitro and in vivo. **a** Western blot analysis of apical supernatants from ALI-cultured NHBE cells exposed to nicotine-containing e-cigarette aerosol (18 mg/mL) or HEPA-filtered air for 0, 8, and 24 h using the ASL 5000 breathing simulator showed increased release of the pro and active forms of MMP-2 and MMP-9, as well as increased pro-MMP-12, following aerosol exposure. **b** Western blot analysis of BAL fluid collected from mice after 4 days of exposure to nicotine-containing e-cigarette aerosol (36 mg/mL) demonstrated elevated pro and active forms of MMP-2 and MMP-9, along with increased pro-MMP-12, compared with air-exposed controls. **c–h** Densitometric quantification confirmed significant increases in MMP-2, MMP-9, and MMP-12 in both NHBE supernatants and mouse BAL fluid. Data are mean ± SD (n = 3–4 replicates per group). One-way ANOVA with Tukey’s multiple comparisons test. *P < 0.05; **P < 0.01; ****P < 0.0001

Primary NHBE cells cultured at the air–liquid interface were exposed to e-cigarette aerosol containing nicotine (18 mg/mL) or HEPA-filtered air for 0, 8, and 24 h using the ASL 5000 breathing simulator. Western blot analysis of apical supernatants demonstrated increased levels of both pro and active forms of MMP-2 and MMP-9, as well as increased pro-MMP-12, following aerosol exposure (Fig. 2a). Densitometric analysis confirmed significant increases in MMP levels in NHBE supernatants (Fig. 2c–e). In parallel in vivo studies, western blot analysis of BAL fluid collected from mice after 4 days of exposure to nicotine-containing e-cigarette aerosol (36 mg/mL) revealed elevated levels of both pro and active forms of MMP-2 and MMP-9, as well as increased pro-MMP-12, compared with air-exposed controls (Fig. 2b). Densitometric quantification confirmed increased MMP expression in BAL fluid (Fig. 2f–h). Together, these findings demonstrate that e-cigarette aerosol exposure induces MMP expression and secretion across both in vitro and in vivo models. The upregulation of MMP-2, MMP-9, and MMP-12 provides a potential mechanistic link to the observed cleavage of junctional proteins such as E-cadherin and subsequent epithelial barrier disruption.

### E-cigarette exposure induces epithelial barrier dysfunction in 16HBE cells in vitro

**Fig. 3:**
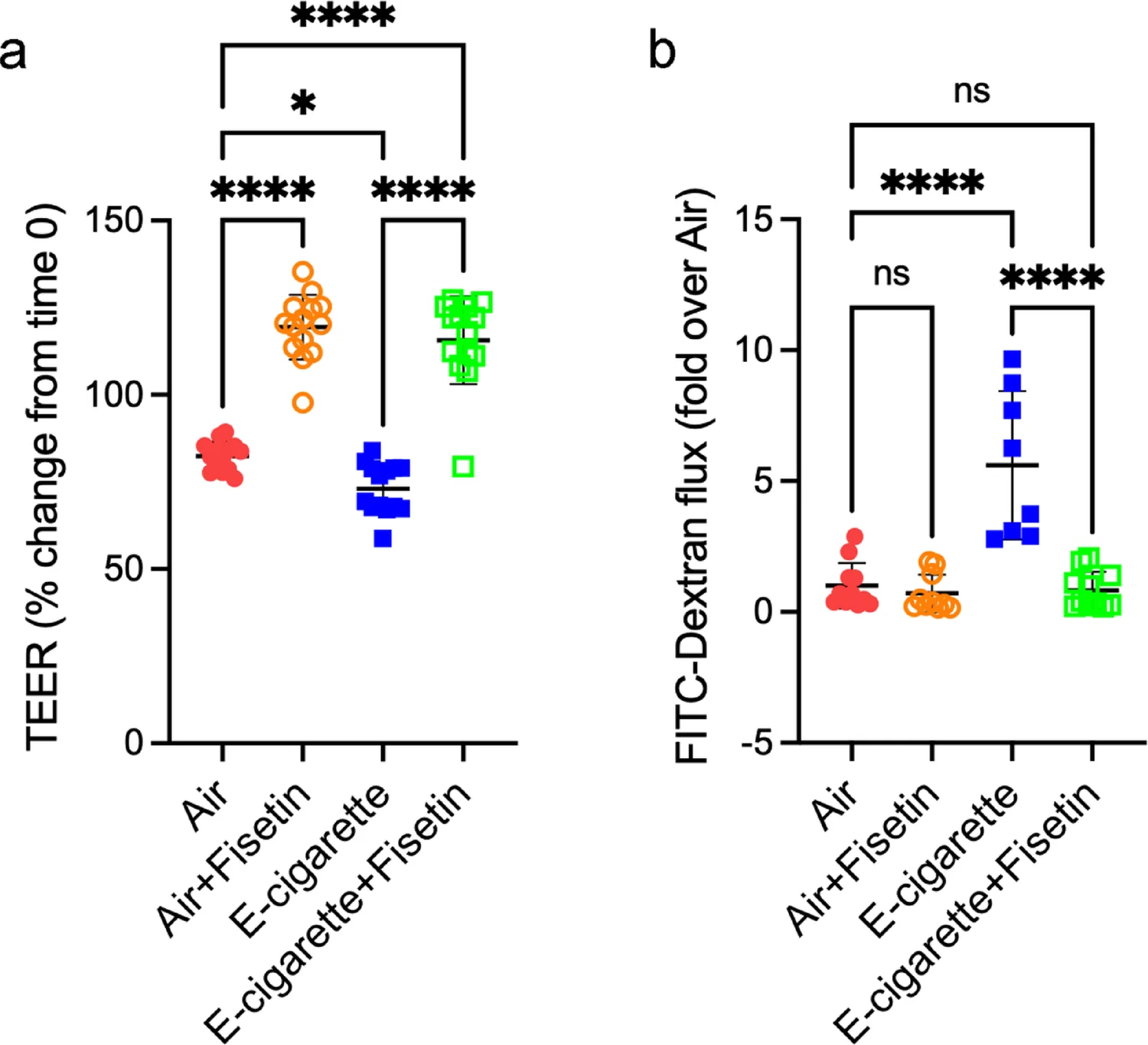
E-cigarette aerosol exposure induces epithelial barrier dysfunction in 16HBE cells that is attenuated by MMP inhibition. 16HBE cells grown to confluence on transwells were exposed to nicotine-containing e-cigarette aerosol (18 mg/mL) or HEPA-filtered air for 0, 8, and 24 h, in the presence or absence of fisetin (20 µM), using the ASL 5000 breathing simulator. **a** TEER measurements (Ω·cm^2^), plotted as percentage of baseline, demonstrated a significant reduction in epithelial resistance following exposure to nicotine-containing aerosols, which was significantly restored by fisetin co-treatment. Data are mean ± SD (n = 14 replicates per group). One-way ANOVA with Tukey’s multiple comparisons test. *P = 0.0363; ***P < 0.0001. **b** FITC-dextran permeability assays showed increased paracellular flux in nicotine-exposed cells relative to air controls, which was significantly attenuated by fisetin treatment. Data are mean ± SD (n = 8–11 replicates per group). One-way ANOVA with Tukey’s multiple comparisons test. **** P < 0.0001

We previously demonstrated that exposure to e-cigarette aerosols increases epithelial permeability and disrupts AJC structure in both in vitro and in vivo models (Beaumont et al. 2025; Raduka et al. 2023). Given the observed increases in sE-cad and MMP expression following e-cigarette aerosol exposure, we next evaluated whether MMP inhibition with the bioflavonoid fisetin could mitigate e-cigarette–induced epithelial barrier dysfunction (Agraval and Yadav 2019). For these experiments, 16HBE cells were grown to confluence on transwells and exposed to aerosolized e-cigarette aerosol containing nicotine (18 mg/mL) or HEPA-filtered air for 0, 8, and 24 h, in the presence or absence of fisetin, using the ASL 5000 breathing simulator. Epithelial barrier integrity was assessed by TEER measurements and FITC-dextran permeability assays. Exposure to nicotine-containing aerosols resulted in a significant reduction in TEER compared with air-exposed controls, indicating compromised epithelial barrier function (Fig. 3a). Co-treatment with 20 µM fisetin significantly restored TEER values in nicotine-exposed cells. Consistent with these findings, FITC-dextran permeability assays demonstrated increased paracellular flux following e-cigarette aerosol exposure, which was significantly attenuated by fisetin treatment (Fig. 3b).

These results indicate that MMP inhibition partially protects against e-cigarette–induced epithelial barrier dysfunction, supporting a role for MMP activity in mediating aerosol-induced permeability changes.

### E-cigarette exposure alters TJ and AJ protein organization

**Fig. 4:**
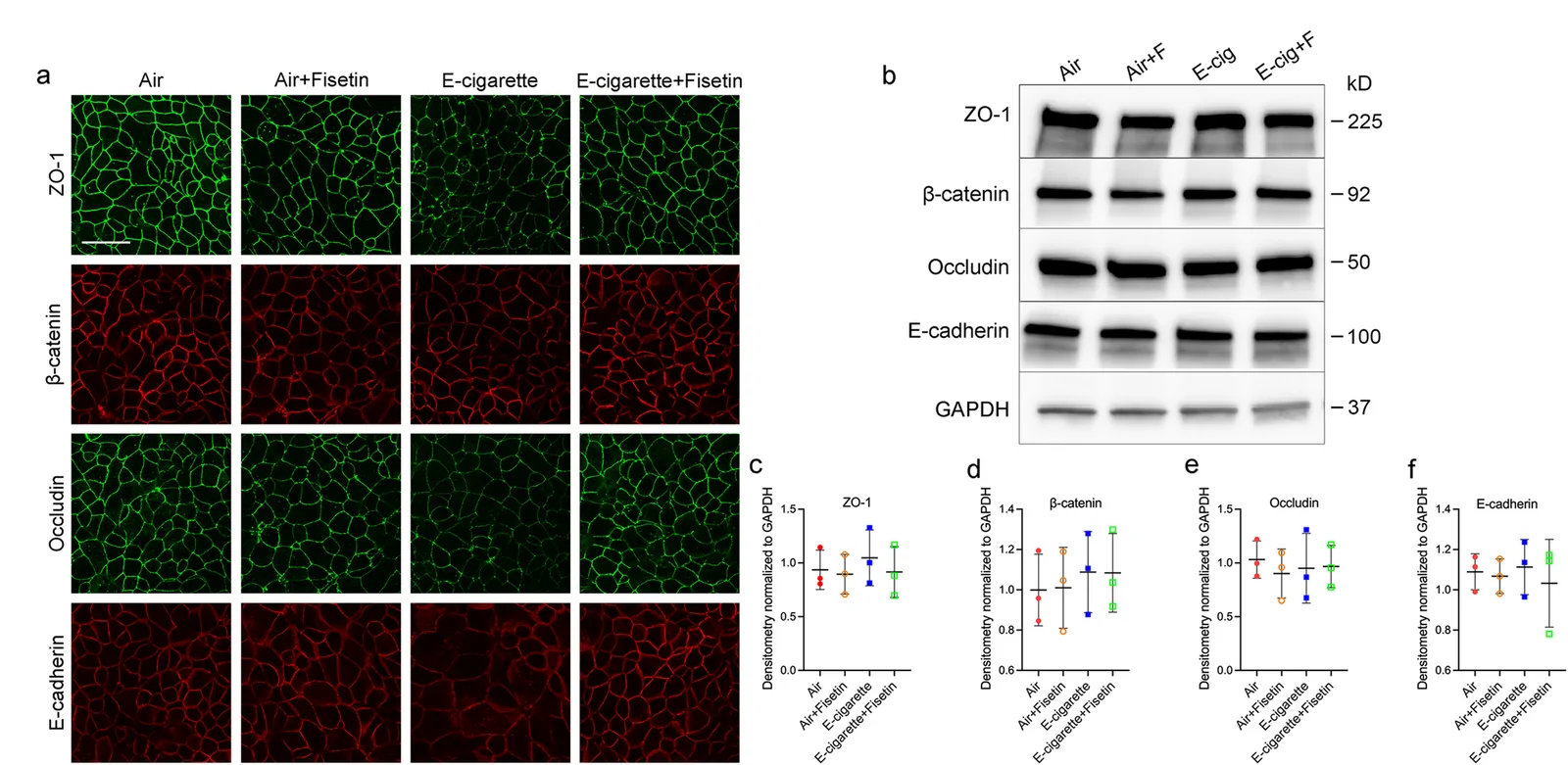
E-cigarette aerosol exposure disrupts tight junction and adherens junction protein organization without altering total protein expression. 16HBE cells grown to confluence on transwells were exposed to nicotine-containing e-cigarette aerosol (18 mg/mL) or HEPA-filtered air for 0, 8, and 24 h, with or without fisetin, using the ASL 5000 breathing simulator. **a** Immunofluorescence staining revealed disrupted and discontinuous localization of TJ proteins (ZO-1, occludin) and AJ proteins (E-cadherin, β-catenin) following nicotine-containing aerosol exposure compared with air-exposed controls. Co-treatment with fisetin preserved junctional organization. Scale bar = 25 µm. Images are representative of three independent experiments. **b–f** Western blot analysis of whole-cell lysates showed no significant changes in total protein levels of ZO-1, occludin, E-cadherin, or β-catenin following e-cigarette aerosol exposure, with or without fisetin. GAPDH served as a loading control. Data are mean ± SD (n = 3 replicates per group). One-way ANOVA with Tukey’s multiple comparisons test. Densitometry of western blot images did not show significant changes between the groups

We next examined the effects of e-cigarette aerosols on airway epithelial TJ and AJ protein organization in the presence or absence of MMP inhibition. 16HBE cells were grown to confluence on transwells and exposed to aerosolized e-cigarette aerosol containing nicotine (18 mg/mL) or HEPA-filtered air for 0, 8, and 24 h, with or without fisetin, using the ASL 5000 breathing simulator. TJ proteins (ZO-1 and occludin) and AJ proteins (E-cadherin and β-catenin) were assessed by immunofluorescence staining and western blot analysis. Immunofluorescence imaging revealed marked disruption of TJ and AJ protein localization following exposure to nicotine-containing aerosols, characterized by disorganized and discontinuous junctional staining compared with the continuous membrane localization observed in air-exposed controls (Fig. 4a). Co-treatment with fisetin reduced junctional disorganization and preserved protein localization. Western blot analysis of total cell lysates showed no significant changes in total protein levels of ZO-1, occludin, E-cadherin, or β-catenin following e-cigarette aerosol exposure, with or without fisetin (Fig. 4b–f).

These findings suggest that e-cigarette aerosols primarily disrupt junctional protein organization rather than overall expression, and that MMP inhibition preserves junctional integrity without altering baseline protein levels.

### MMP inhibition reduces e-cigarette–induced sE-cad release

**Fig. 5:**
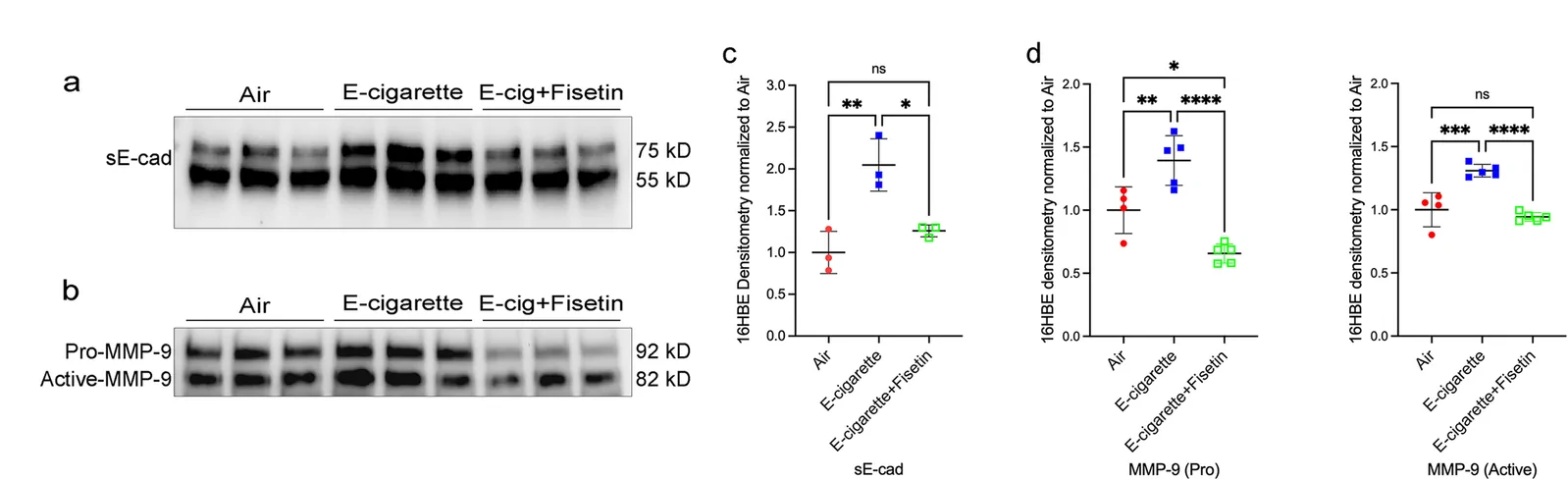
MMP inhibition attenuates e-cigarette aerosol–induced E-cadherin cleavage and selectively reduces MMP-9 expression. **a** Western blot analysis of apical supernatants from 16HBE cells grown on transwells exposed to nicotine-containing e-cigarette aerosol (18 mg/mL) or HEPA-filtered air for 0, 8, and 24 h, with or without fisetin, demonstrated significant increases in sE-cad following aerosol exposure, which were significantly attenuated by fisetin treatment. **c** Densitometric quantification confirmed reduced sE-cad release following MMP inhibition. **b**, **d** Western blot analysis and densitometric quantification demonstrated a significant reduction in MMP-9 expression with fisetin treatment. Data are presented as mean ± SD (n = 3 independent experiments per group). Statistical analysis was performed using one-way ANOVA with Tukey’s multiple comparisons test. *P < 0.05; **P < 0.01; ***P < 0.001; ****P < 0.0001

To determine whether MMP activity directly contributes to E-cadherin cleavage, we measured sE-cad levels in apical supernatants following MMP inhibition. 16HBE cells grown on transwells were exposed to nicotine-containing e-cigarette aerosol (18 mg/mL) or HEPA-filtered air for 0, 8, and 24 h, with or without fisetin, using the ASL 5000 breathing simulator. sE-cad levels were assessed by western blot analysis of apical supernatants. In 16HBE models, fisetin treatment significantly reduced the elevated sE-cad levels induced by e-cigarette aerosol exposure (Fig. 5a). Densitometric quantification confirmed decreased sE-cad expression in cells exposed to fisetin (Fig. 5c). Consistent with these findings, fisetin treatment was associated with a significant reduction in MMP-9 expression (Fig. 5b, d), whereas MMP-2 and MMP-12 expression were not significantly altered (not shown). Collectively, these findings demonstrate a mechanistic role for MMPs—particularly MMP-9—in e-cigarette–induced epithelial injury and support the potential of pharmacologic MMP inhibition to protect against junctional disruption and epithelial barrier dysfunction.

## Discussion

### Summary of findings

This study demonstrates that e-cigarette aerosols promote E-cadherin cleavage and increase MMP expression in both murine exposure models and human airway epithelial cells. These findings reveal a mechanistic pathway through which e-cigarettes compromise epithelial barrier integrity. These results place e-cigarette aerosols within a broader spectrum of inhaled toxicants known to destabilize epithelial adhesion complexes. By identifying a defined MMP–E-cadherin axis, our study provides a mechanistic framework linking exposure to structural injury and offers a biologically plausible explanation for the epithelial vulnerability observed in e-cigarette users.

### Mechanistic insights

To understand how e-cigarette aerosols impair epithelial barrier function, we examined the role of E-cadherin cleavage and MMP activation. E-cadherin plays a central role in epithelial physiology, maintaining barrier cohesion through incorporation into the AJC (Gumbiner 2005). E-cadherin has an extracellular domain, a single-pass transmembrane segment, and a conserved cytoplasmic tail (Pokutta and Weis 2007). The extracellular portion is susceptible to proteolytic cleavage via ectodomain shedding, generating a soluble 60–80 kDa N-terminal fragment (sE-cad) from the full-length 120 kDa protein (Nawijn et al. 2011). Given its role in junctional stability and homeostasis (Nava et al. 2013; Nawijn et al. 2011), ectodomain cleavage disrupts paracellular integrity and compromises barrier function.

Our findings demonstrate that e-cigarette aerosols induce E-cadherin cleavage through upregulation of MMPs. These MMPs mediate extracellular matrix degradation and E-cadherin processing in multiple inflammatory and injury contexts (David and Rajasekaran 2012; Lynch et al. 2010; Nava et al. 2013; Ray et al. 2023). We selected MMP-2, MMP-9, and MMP-12 for investigation because these enzymes have been consistently implicated in pathological lung remodeling and inflammation. All three MMPs degrade components of the extracellular matrix and are upregulated in human lung diseases, including COPD and emphysema, particularly in response to cigarette smoke exposure (Cabral-Pacheco et al. 2020; Greenlee et al. 2007). MMP-9 is of particular interest due to its established role in E-cadherin cleavage, a process implicated in epithelial barrier dysfunction (Lialios and Alimperti 2025). Collectively, these enzymes represent key mediators of smoke-induced lung pathology, making them highly relevant targets for evaluating their association with e-cigarette exposure. Elevated sE-cad has been reported in pulmonary fibrosis, COPD, asthma, and infections, where it correlates with epithelial disruption (Frank and Hostetter 2007; Lialios and Alimperti; Samuels et al. 2023). Our study extends this association to vaping, establishing MMP-dependent E-cadherin cleavage as a key mechanism of epithelial injury.

Importantly, nicotine emerges as a central driver of MMP induction and subsequent E-cadherin cleavage in the context of e-cigarette exposure. Nicotine’s context-dependent immunologic effects include protease activation, and our findings demonstrate that nicotine significantly contributes to vaping-induced airway barrier dysfunction via MMP-dependent pathways, highlighting its pivotal role in epithelial injury.

Given this mechanistic link, we evaluated whether inhibiting MMP activity could prevent sE-cad generation and epithelial dysfunction. Treatment with the broad-spectrum MMP inhibitor fisetin markedly reduced E-cadherin cleavage in exposed bronchial epithelial cells, confirming that shedding is MMP-dependent. Interestingly, fisetin selectively reduced MMP-9 expression without significantly altering MMP-2 or MMP-12 levels, suggesting that MMP-9 may be a dominant driver of E-cadherin cleavage in this context. Similar protective effects of MMP inhibition have been reported in other models of epithelial injury (Frank and Hostetter 2007; Haderer et al. 2022), although the clinical utility of broad-spectrum inhibitors remains limited.

Beyond sE-cad elevation, exposure to nicotine-containing aerosols caused pronounced disruption of AJ and TJ organization, producing discontinuous, fragmented, and disorganized staining patterns. Fisetin partially restored normal junctional architecture, indicating that MMP-driven E-cadherin cleavage contributes to downstream destabilization of the AJC. These structural changes translated into impaired barrier function, reflected by decreased TEER and increased FITC-dextran flux.

Notably, total AJ and TJ protein levels remained stable despite these alterations, suggesting that e-cigarette exposure disrupts barrier function primarily through junctional mislocalization and cytoskeletal remodeling rather than reduced protein abundance—a pattern consistent with acute RSV exposure models (Rezaee et al. 2013, 2011). Restoration of TEER and paracellular permeability with fisetin further supports a model in which MMP activation and sE-cad generation act upstream of vaping-induced epithelial barrier dysfunction.

### Clinical and translational relevance

Our findings support a model in which e-cigarette aerosols activate MMPs, promote E-cadherin cleavage, and compromise epithelial barrier integrity. sE-cad may serve as a measurable indicator of vaping-related epithelial injury. While not disease-specific, elevated sE-cad has been observed across cancers, infections, organ failure, and metabolic disease, where it correlates with epithelial barrier loss (David and Rajasekaran 2012; Grabowska and Day 2012; Martin and Jiang 2009; Repetto et al. 2014; Smallcombe et al. 2019; Symowicz et al. 2007). Its detectability in serum and urine makes it a practical candidate biomarker (Grabowska and Day 2012; Kazem et al. 2023). Oxidative stress markers such as 8-OHdG and 8-isoprostane have been proposed as potential indicators in individuals with e-cigarette or vaping product use-associated lung injury (EVALI) (Podguski et al. 2022). Given that sE-cad is relevant to multiple disease states, it could serve as a complementary biomarker alongside other markers, particularly for detecting subclinical epithelial injury before symptoms arise. In human studies, direct therapeutic MMP inhibition has been limited by toxicity and poor specificity (David and Rajasekaran 2012). Alternative approaches—such as neutralizing sE-cad, modulating upstream pathways that regulate MMP expression, or selectively targeting specific MMP isoforms—may offer more viable translational strategies. Studies measuring sE-cad in human e-cigarette users are needed to determine its utility as a biomarker and to guide therapeutic development.

### Limitations and future directions

Several limitations warrant consideration. Although our data identify MMP activation as a key mediator of E-cadherin cleavage following e-cigarette aerosol exposure, upstream pathways driving MMP induction were not defined and may involve oxidative stress–responsive signaling (e.g., NF-κB, AP-1), receptor-mediated effects of nicotine, or direct responses to other aerosol constituents. Defining these pathways will be important for developing more selective intervention strategies.

Although MMP-7 is a known mediator of E-cadherin cleavage, we were unable to reliably detect its expression in our models using available antibodies; future studies using alternative detection approaches will be needed to determine its role in e-cigarette–induced epithelial injury.

While increases in pro- and active forms of MMPs were observed, direct measurement of enzymatic activity was not successful under our experimental conditions. Future studies incorporating optimized activity assays (e.g., zymography or fluorometric approaches) will be important to further define the functional role of specific MMPs in mediating E-cadherin cleavage.

The functional role of sE-cad was not directly examined. Prior studies suggest that sE-cad may disrupt junctional adhesion, promote epithelial–mesenchymal transition, and perpetuate barrier instability (Lialios and Alimperti 2025; Nava et al. 2013). Additionally, downstream processing of the membrane-bound C-terminal E-cadherin fragment—potentially involving γ-secretase–mediated cleavage and nuclear translocation—was not assessed and may influence epithelial gene expression (Nava et al. 2013).

An additional limitation is the absence of a nicotine-free aerosol control, which precludes full separation of nicotine-specific effects from those of other aerosol constituents. Although prior work from our group demonstrated that PG:VG solvents independently affect airway epithelial function (Raduka et al. 2023), the use of commercially available e-liquids in this study limited our ability to obtain reliable nicotine-free formulations. Notably, products labeled as “nicotine-free” may still contain detectable nicotine, reducing their utility as true negative controls, and standardized nicotine-free PG:VG formulations suitable for aerosolization were not consistently available (Goniewicz et al. 2015). Future studies using well-characterized, validated nicotine-free aerosols will be important to more precisely define the relative contributions of nicotine and non-nicotine components.

Another limitation of this study is the use of a 3rd generation ENDS device, which produces aerosol characteristics that may differ from earlier-generation devices and newer pod-based systems in terms of power output, aerosol composition, and nicotine delivery. As such, these findings may not fully generalize across all e-cigarette device types, and future studies comparing multiple device generations are warranted.

An additional consideration is that while our data support a role for MMP-mediated E-cadherin cleavage, experiments using MMP inhibitors at physiologically relevant concentrations were limited by experimental constraints and thus should be interpreted cautiously. Preliminary data generated under these conditions demonstrate a consistent directional effect supporting the proposed mechanism but will require further validation in fully replicated studies.

Finally, our findings are based on a single nicotine concentration and acute exposure conditions, which preclude assessment of dose–response and temporal effects. Chronic and repeated vaping exposures may have cumulative effects on junctional regulation and airway remodeling, necessitating longer-term in vivo and human studies.

## Conclusion

E-cigarette aerosols induce a “leaky” epithelial barrier via MMP-mediated E-cadherin cleavage, increased sE-cad production, disruption of AJ and TJ organization, and impaired epithelial permeability. These findings reveal a previously unrecognized pathogenic mechanism of e-cigarette–associated airway injury, implicating MMP activity and E-cadherin ectodomain shedding as central drivers of barrier dysfunction. sE-cad may serve as a potential biomarker, and MMP-targeted interventions to reverse barrier defects warrant further investigation. Additional studies are needed to define upstream signaling pathways, validate sE-cad in human cohorts, and determine the long-term impact of sustained E-cadherin cleavage on airway physiology.

## Data Availability

Data will be made available upon reasonable request.

## Abbreviations

16HBE: 16HBE14o- human bronchial epithelial
AJ: Adherens junction
AJC: Apical junctional complex
ALI: Air liquid interface
ATCC: American Type Culture Collection
BAL: Bronchoalveolar lavage
BSA: Bovine serum albumin
E-cigarette: Electronic cigarette
E-liquid: Electronic liquid
ENDS: Electronic nicotine delivery system
EVALI: E-cigarette or vaping product use-associated lung injury
FBS: Fetal bovine serum
FITC: Fluorescein isothiocyanate
GAPDH: Glyceraldehyde-3-phosphate dehydrogenase
GM-6001: Ilomastat
IHC: Immunohistochemistry mAbs: primary monoclonal antibodies
MMPs: Matrix metalloproteinases
NHBE: Primary normal human bronchial epithelial cells
pAbs: Polyclonal antibodies
PBS: Phosphate -buffered saline
PFA: Paraformaldehyde
PVDF: Polyvinylidene difluoride
RSV: Respiratory syncytial virus
RT: Room temperature
sE-cad: Soluble E-cadherin
TEER: Transepithelial electrical resistance
TJ: Tight junction
WBC: Whole-body mouse exposure chamber
Z-fix: Zinc buffered formalin
ZO-1: Zonula occludens-1
